# Presence and diversity of *Salmonella *isolated from layer farms in central Ecuador

**DOI:** 10.12688/f1000research.18233.2

**Published:** 2019-04-09

**Authors:** Gabriela A. Salazar, Ricardo Guerrero-López, Liliana Lalaleo, Diana Avilés-Esquivel, Christian Vinueza-Burgos, William Calero-Cáceres

**Affiliations:** 1 UTA RAM OneHealth Group, Faculty of Agricultural Sciences, Universidad Técnica de Ambato, Cevallos, Ecuador; 2 Facultad de Medicina Veterinaria y Zootecnia, Universidad Central del Ecuador, Quito, Ecuador

**Keywords:** Salmonella, Layer Poultry, Ecuador, Serovars.

## Abstract

**Background:** Given the considerable role played by *Salmonella* in the incidence of food contamination, around the world, surveillance of this infection is prioritized by both food producers and health care authorities. Data remains insufficient concerning the prevalence of *Salmonella* in poultry systems in Ecuador and in Latin America in general.

**Methods:** In this study, we evaluated the presence and diversity of *Salmonella *serovars in samples taken from 21 layer farms and backyard layers in central Ecuador during August-November 2017. *Salmonella *was isolated following standardized methods (ISO 6579) and the serovar determination was carried out by PCR.

**Results: **A significant presence of *Salmonella* was detected in the 21 farms evaluated, with a frequency of 76% (95% confidence interval (CI): 53-92) in environmental surfaces, 33% (95%CI: 15-57) in pooled cloacal swabs from layer hens, 33% (95% CI: 13–59) on feed samples, and 10% (95%CI: 1-30) in backyard layer feces from traditional local markets. The dominant serovars detected were *S. *Infantis and *S. *Typhimurium.

**Conclusions:** This study forms a basis for further surveillance of *Salmonella *serovars in layer farms in central Ecuador.

## Introduction

The genus *Salmonella* is considered a leading cause of foodborne illnesses around the world ( WHO, 2017). These bacteria are among the most significant agents of food and water poisoning in the United States and Europe ( Bäumler *et al.*, 2000; Callejón *et al.*, 2015; Varma *et al.*, 2005). Globally, it is estimated that 93.8 million cases of *Salmonella*-related gastroenteritis occur annually, resulting in 155,000 deaths ( Majowicz *et al.*, 2010). In Ecuador, typhoid, together with paratyphoid fever, causes around 1,500 hospitalizations per year, while non-typhoidal salmonellosis leads to more than 2,000 hospitalizations over the same period. These infections have accounted for approximately 25% of total reported gastrointestinal illnesses in recent years ( MSP, 2018). *Salmonella* represents a complex and diverse genus, but only a small number of serovars are involved in human infections ( Issenhuth-Jeanjean *et al.*, 2014; Thiennimitr *et al.*, 2012).

*Salmonella* detection and the investigation of foodborne outbreaks is of maximum importance to public health, which has resulted in the establishment of epidemiological surveillance programs in many developed countries ( EFSA, 2013). These measures provide information about endemic *Salmonella*-serovar patterns, outbreaks, temporal trends and the monitoring of control actions ( CDC, 2018). The principal reservoir of *S. enterica* is the intestinal tract of livestock, representing one of the main sources of infection for humans ( Antunes *et al.*, 2016). Despite this epidemiological and economic importance, in South America there is a paucity of information concerning *Salmonella* in poultry systems ( Alexandre *et al.*, 2000; Donado-Godoy *et al.*, 2012). Research conducted in 2016 pertaining to broiler chicken farming in Ecuador indicated the presence of *Salmonella* serotypes *S.* Infantis *, S.* Enteritidis and *S.* Corvallis ( Vinueza-Burgos *et al.*, 2016). However, among layer hens, no information about the prevalence or diversity of *Salmonella* has been reported in this country nowadays. The purpose of this study was to estimate the presence and diversity of *Salmonella* bacteria present in 21 layer farms in central Ecuador.

## Methods

### Samples

Samples were collected between August–November 2017 from different layer farms in central Ecuador (Tungurahua and Cotopaxi provinces), which accounts for around 60% of egg production in the country. A total of 21 farms (>1,000 birds) in Latacunga, Cevallos, Quero and Ambato (all Ecuador) were sampled, based on their willingness to provide verbal consent for this study (verbal consent was obtained over written consent owing to the farmers’ reluctance to sign their names, as they perceived this could be used to identify them). Further details for each site can be found in Dataset 1 ( Calero-Cáceres, 2019a)). One laying hen house per farm was selected. The following samples were collected in each house: 21 pooled cloacal swabs (10 cloacal swabs per pool); 21 manure drag swabs (environmental swabs, 1 per business); 21 caecum content samples (1 layer per farm). To evaluate the potential risk from contaminated feed, 18 composite samples were taken from farmyards (18 of the 21 farms consented verbally to having these samples taken). Additionally, 21 fecal samples from backyard layers were sampled in traditional local markets. All samples were transported in an icebox at 3–5°C within 2 hours of collection for bacterial isolation. The experiment was performed under supervision of the ethical committee of the Faculty of Agricultural Sciences, Universidad Técnica de Ambato.

### Detection of *Salmonella*

*Salmonella* was isolated following standardized methods (ISO 6579) ( ISO, 2017). The samples were pre-enriched in buffered peptone water (Oxoid, Basingstoke, England) and then incubated at 37±1°C for 18 h ± 2 h. Next, Rappaport Vassiliadis Soy Broth (RVS Broth) (Merck Millipore, Darmstadt, Germany) was used a selective medium, being inoculated with the pre-enriched culture and incubated at 41.5±1°C for 24±3 h. One loopful of the selective enrichment medium was streaked onto xylose lysine deoxycholate agar (XLD agar) (Becton Dickinson GmbH, Heidelberg, Germany) and incubated at 37±1°C for 24±3 h. Presumptive *Salmonella* isolates (identified as red/yellow colonies with a black center) were purified in Mac Conkey agar (Merck, Darmstadt, Germany) and incubated at 37±1°C for 24 h. Isolates were Gram stained and the following biochemical tests were performed for confirmation the genus *Salmonella*: a catalase test using 30% hydrogen peroxide (Merck Millipore, Darmstadt, Germany); triple sugar iron agar test (TSI) (Becton Dickinson GmbH, Heidelberg, Germany), Simmons citrate agar test (Merck, Darmstadt, Germany), Christensen urea agar test (Britania Lab., Buenos Aires, Argentina), and indole reaction using tryptone water (Merck, Darmstadt, Germany) and Kovac’s reagent (Sigma Aldrich, St. Louis, USA). One isolate per positive sample was selected and cryopreserved using overnight growth in LB broth (Sigma Aldrich, St. Louis, USA) supplemented with 30% glycerol (Merck Millipore, Darmstadt, Germany) and maintained at -80°C until analysis.

### Serovar determination by PCR

PCR assays were performed to identify the genes under specific conditions ( Table 1). One virulence gene related with fimbrial cluster ( *bcfC*) was evaluated as target of *S. enterica* specie ( Zhu *et al.*, 2015). For serovars and biotypes: A modification methylase gene that are specific of *S.* Infantis (M.SinI) ( Ranjbar *et al.*, 2017). 23S rRNA gene associated to *S.* Typhi ( *sty*) ( Pui *et al.*, 2011). A flagellin gene that show specificity to *S.* Typhimurium ( *fliC*)( Pui *et al.*, 2011). Gallinarum biotypes were identified by *steB* fimbrial and *rhs* locus ( Zhu *et al.*, 2015). Specific DNA difference fragment (SdfI) served as target to distinguish *S.* Enteritidis from other serovars ( Agron *et al.*, 2001). And a putative membrane protein ( *gly*) to identify *S.* Kentucky ( Zhu *et al.*, 2015). DNA was extracted from overnight cultures in Casein-Peptone Soymeal-Peptone Broth (Merck, Darmstadt, Germany) as described by Muniesa *et al*. (2004). Approximately ≈50 ng/reaction resulting from the thermal shock of bacterial suspensions (>10 ^7^ UFC/ml) diluted in sterile ddH _2_0 was used as template for the PCR reactions. Amplifications were carried out as follows: initial denaturation at 95°C for 1 min; 35 cycles of 95°C for 30 s, annealing temperature according to Table 1 for 30 s, extension at 72°C for 1 min; and a final elongation step at 72°C for 7 min. The following reference *Salmonella* strains were used as positive controls for the six serovars under investigation: *S.* Enteritidis UNIETAR 1, *S.* Gallinarum b. Gallinarum NCTC 13346, *S.* Gallinarum b. Pullorum ATCC 19945®, *S.* Typhi ATCC® 19430, *S.* Typhimurium ATCC® 26930 and *S.* Infantis UNIETAR 3CT7.

**Table 1.** Oligonucleotides used in this study.

Organism	Name and direction	5’ to 3’ sequence	Gene	Annealing temperature (°C)	Amplicon size (bp)	References
Enteritidis	sdf-F	TGT GTT TTA TCT GAT GCA AGA G	sdf locus	56	293	( Agron *et al*., 2001)
	sdf-R	CGT TCT TCT GGT ACT TCA GAT GAC				
Enterica	bcfC-F	GGG TGG GCG GAA AAC TAT TTC	bcfC	56	993	( Zhu *et al*., 2015)
	bcfC-R	CGG CAC GGC GGA ATA GAG CAC				
Infantis	M. SinI F	CAC AAT GAA CGT GGT GAA GG	M.Sin I	56	184	( Ranjbar *et al*., 2017)
	M. SinI R	TGA ACT ACG TTC GTT CTT CTGG				
Gallinarum biotype Gallinarum	steB-F	TGT CGA CTG GGA CCC GCC CGC CCG C	steB	56	636	( Pugliese *et al*., 2011)
	steB-R	CCA TCT TGT AGC GCA CCA T				
Gallinarum biotype Pullorum	rhs-F	TCG TTT ACG GCA TTA CAC AAG TA	rhs locus	56	402	( Zhu *et al*., 2015)
	rhs-R	CAA ACC CAG AGC CAA TCT TAT CT				
Typhi	sty-1	TGC CGG AAA CGA ATC T	23S rRNA gene	53	300	( Zhu *et al*., 1996)
	sty-2	GGT TGT CAT GCC AAT GCA CT				
Typhimurium	Fli15	CGG TGT TGC CCA GGT TGG TAA T	fliC gene	53	620	( Pui *et al*., 2011)
	Typ04	ACT GGT AAA GAT GGC T				

The reaction mixture contained: 12.5 µl of DreamTaq Green PCR Master Mix (Thermo Fisher Scientific, Massachusetts, USA), 0.5 µl of each primer (30 µM stock), 9 µl of nuclease-free water (Thermo Fisher Scientific, Massachusetts, USA), and 2.5 µl of crude DNA were used. PCRs were performed with an Applied Biosystems SimplyAmp Thermal Cycler (Thermo Fisher Scientific, Massachusetts, USA). A total of 10 µl of each PCR product were analyzed by agarose gel electrophoresis and stained using Sybr® Safe DNA Gel Stain (Invitrogen, Carlsbad, USA).

## Results

### Assessing the presence of *Salmonella*

Overall, 31 out of 34 isolates which showed phenotypic characteristics in accordance with *Salmonella* were confirmed by PCR as *S. enterica* ( Table 2). Of the 21 farms, 16 (76%, 95%CI: 53-92) showed the presence of *Salmonella* on environmental surfaces, and *Salmonella* bacteria were isolated in 7 pooled cloacal swabs (33%, 95%CI: 15–57) and in 6 of 18 composite feed samples (33%, 95%CI: 13–59). Feces from backyard layers showed the presence of *Salmonella* in 2 of 21 samples (10%, 95%CI: 1–30). Dataset 1 shows the location of each sample and the presence or absence of *Salmonella* ( Calero-Cáceres, 2019a)

**Table 2.** *Salmonella*-positive samples in relation to evaluated matrices.

Location	Sample	Matrix	Samples, n	Positive samples, n (%)
Farms	Feed	Composite feed	18	6 (33%)
	Environment	Environmental swabs	21	16 (76%)
	Animal	Laying hen cloacal swab (composite)	21	7 (33%)
Local markets	Environment	Backyard poulty feces	21	2 (10%)

### Determining presence of serovars

**Figure 1.**
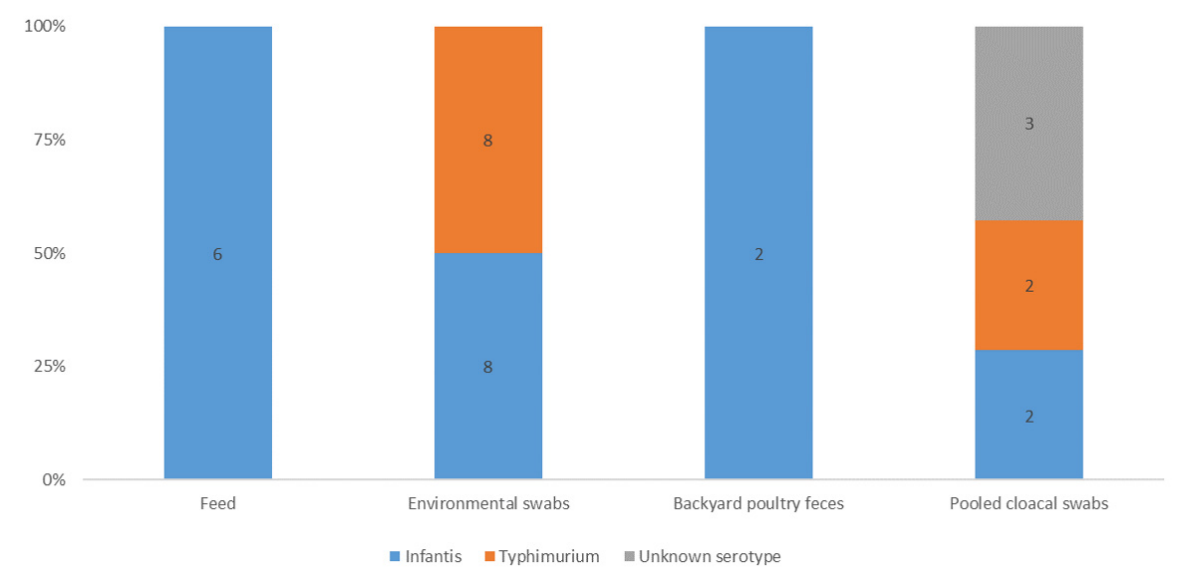
Distribution of *Salmonella* serovars according to the evaluated matrices.

Regarding to the presence of individual serovars ( Figure 1), the highest occurrence was of *S.* Infantis, detected in 58% (18/31) of isolates, followed by *S*. Typhimurium, present in 32% of samples (10/31) ( Supplementary Figure 1 ( Calero-Cáceres, 2019c)). The serovar of 10% of *Salmonella* isolates (3/31) were unable to be serotyped by the panel of tests. In poultry feed samples, *S.* Infantis was present in 100% (6/6), while in *S. enterica* isolated from manure drag swabs, *S.* Infantis was detected in 50% (8/8) and *S.* Typhimurium in 50% (8/8). In backyard poultry feces from local markets, *S.* Infantis was detected in 100% of positive samples (2/2). The most heterogeneous diversity of *Salmonella* serotypes was observed in pooled cloacal swabs, with 2 *S.* Infantis, 2 S. Typhimurium and 3 *S. enterica* consisting of serovars not covered within the panel. Dataset 1 lists the identity of the serovar taken from each sampling location ( Calero-Cáceres, 2019a). Dataset 2 shows PCR gels for confirmation of *Salmonella* serovars ( Calero-Cáceres, 2019b).

## Discussion

The overall results showed a significant presence of *Salmonella* in layer farms in the central Ecuador region, one of the main sources of egg production in the country, with a detection of at least one sample positive per farm in the 76% of the evaluated sites. This result is similar to that reported in Colombia (65%) ( Donado-Godoy *et al*., 2012), and considerably higher than the presence of *Salmonella* in broiler chicken farms in northern Ecuador (15.9%) ( Vinueza-Burgos *et al*., 2016).

The notable presence of *Salmonella* in poultry feed suggests that this may constitute a potential reservoir of this bacteria for poultry systems in the evaluated region, and consequently, a potential route of infection and colonization of poultry and subsequent entry into the food chain ( Fink-Gremmels, 2012; Jones, 2011). In recent research conducted in an integrated poultry farm in Ecuador, 4.1% (8/194) of samples were positive for *Salmonella*, particularly in animal-based feed ( Villagómez Estrada *et al*., 2017). Therefore, the accurate detection of this pathogen in feed is necessary in order to identify the critical points of contamination (facilities, raw materials, transport, storage, producers), that one may apply effective measures to reduce the risk of transmission.

The finding of only *S.* Infantis in poultry feed may be attributed to the high level of persistence of this serovar over time in poultry feed, which in turn results in its considerable presence in Ecuadorian broiler chicken ( Andino *et al.*, 2014; Vinueza-Burgos *et al.*, 2016). Although the origin of this serovar in feed it is not well defined, studies have pointed to cross-contamination with feces, persistent contamination of storage bins and surfaces, and poor ingredient selection as main causes of feed contamination with *Salmonella* ( Davies & Wray, 1997; Huss *et al.*, 2018; Laban *et al.*, 2014). Genomic tools, such as multilocus sequence typing, BOX-PCR, (GTG)5-PCR or whole-genome sequencing may help to identify the origin of feed contamination in a larger study.

Drag swabs revealed the presence of two different serovars in the sampled poultry farms: Infantis and Typhimurium. These non-typhoidal *S. enterica* serovars are commonly associated with poultry systems and are linked to outbreaks of foodborne illness ( Anderson *et al.*, 2016; Pui *et al.*, 2011). Serovars vary in their persistence over time and geographic distribution around the world ( Hendriksen *et al.*, 2011), making further studies desirable in order to evaluate the variations in serovar persistence in the locations sampled in this research.

In backyard layer feces sampled at local markets, *S.* Infantis alone was detected (2/2), but further evaluation of *Salmonella* serovars in backyard flocks is recommended in order to improve surveillance of this potential source of salmonellosis ( Behravesh *et al.*, 2014). In pooled cloacal swabs, the serovars detected were Infantis (2/7), Typhimurium (2/7) and unidentified serovars (3/7). Infantis and Typhimurium serovars are commonly detected in poultry around the world ( Foley & Lynne, 2008; Foley *et al.*, 2011). Complementary analysis by serotyping the unclassified serovars is necessary in order to identify *Salmonella* bacteria not covered by the panel.

Data remains insufficient concerning the prevalence of *Salmonella* in poultry systems in Ecuador and in Latin America in general. The coordination of similar future studies may provide a starting point for surveillance of zoonotic bacteria within a defined public health area, leading to an improvement in policies and safe practices in the food industry. Such studies may reduce the risk of infection and establish protocols for corrective measures to be implemented in key upstream points of the chain as indicated by the data.

At the same time, the intervention of public health authorities may be required in order to ensure the participation of a fully representative range of poultry businesses in future research. This study depended on the voluntary consent of farm owners to providing samples, which may have led to selection bias. In order to establish a thorough program of surveillance, all potential sources of *Salmonella* infection in the region need to be made accessible to researchers.

These findings show a significant presence of *Salmonella* in layer farms in the central zone of Ecuador. The predominant serovars are *S.* Infantis and *S.* Typhimurium, typified by PCR. The principal source of infection could be related with poultry feed. From a public health perspective, it is necessary to establish adequate surveillance of *Salmonella*, including protocols covering biosecurity practices, antibiotic usage and random sampling programs.

## Data availability

### Underlying data

Figshare: Dataset 1. Origin, biochemical tests, PCR results and serovar of *Salmonella* isolates. https://doi.org/10.6084/m9.figshare.7726163.v2 ( Calero-Cáceres, 2019a)

Figshare: Dataset 2. PCR results of bcfC, fliC and M.SinI genes. https://doi.org/10.6084/m9.figshare.7726217.v2 ( Calero-Cáceres, 2019b)

### Extended data

Figshare: Supplementary figure 1. Multiplex PCR amplification of serovar-specific genes. a) *bcfC* and *mSinI* fragments ( *S.* Infantis), b) *bcfC* and *fliC* fragments ( *S.* Typhimurium).

https://doi.org/10.6084/m9.figshare.7732934.v1 ( Calero-Cáceres, 2019c.)

Data are available under the terms of the Creative Commons Zero "No rights reserved" data waiver (CC0 1.0 Public domain dedication).
