# Supplementary material for: Presence and diversity of Salmonella isolated from layer farms in central Ecuador
Source: F1000Res. 2019 Apr 9;8:235. Originally published 2019 Feb 28. [Version 2] doi: 10.12688/f1000research.18233.2 (PMC6480948; doi:10.12688/f1000research.18233.2)
Supplement: Peer review history

# Peer review: Reviewer response for version 2

**DOI:** 10.5256/f1000research.20651.r46971

**Published:** 2019-04-17

**Authors:** Tania Gaviria Cantín

**Affiliations:** 1 Departamento de Ciencias Naturales, Exactas y Estadística. Facultad de Ciencias Básicas, Universidad de Santiago de Cali, Cali, Colombia

I have read this submission. I believe that I have an appropriate level of expertise to confirm that it is of an acceptable scientific standard.

# Peer review: Reviewer response for version 2

**DOI:** 10.5256/f1000research.20651.r46970

**Published:** 2019-04-09

**Authors:** Eloy Gonzalez-Gustavson

**Affiliations:** 1 Facultad de Medicina Veterinaria, Universidad Nacional Mayor de San Marcos, Lima, Peru

I agree with the changes.

I have read this submission. I believe that I have an appropriate level of expertise to confirm that it is of an acceptable scientific standard.

# Peer review: Reviewer response for version 1

**DOI:** 10.5256/f1000research.19945.r45085

**Published:** 2019-03-21

**Authors:** Eloy Gonzalez-Gustavson

**Affiliations:** 1 Facultad de Medicina Veterinaria, Universidad Nacional Mayor de San Marcos, Lima, Peru

The scientific names in the manuscript need to be reviewed.

 The author use incorrectly the terms prevalence and incidence as synonyms.

 Incidence is defined as the presence of new cases of an illness over a period of time. The manuscript does not correspond to an incidence study.

 I would like to see more information in the manuscript about the design to consider the results as prevalence. I have not found details about the representativeness of the sampling (sample size to get a prevalence) or if they were obtained randomly. If the method does not satisfy the requirement to be considered as prevalence, I recommend replacing the term prevalence by frequency, presence or proportion.

 The confidence interval in one case was incorrectly estimated.

 "Feces from backyard layers showed the presence of *Salmonella* in 2 of 21 samples (10%, 95%CI: 0–22)"

 This values have to be calculated based on binomial distribution. Actually in the manuscript, the confidence interval was estimated based on normal or t distribution and 0 was included as lower interval.

I have read this submission. I believe that I have an appropriate level of expertise to confirm that it is of an acceptable scientific standard, however I have significant reservations, as outlined above.

# Peer review: Reviewer response for version 1

**DOI:** 10.5256/f1000research.19945.r45084

**Published:** 2019-03-11

**Authors:** Tania Gaviria Cantín

**Affiliations:** 1 Departamento de Ciencias Naturales, Exactas y Estadística. Facultad de Ciencias Básicas, Universidad de Santiago de Cali, Cali, Colombia

**Title**

 I consider that the title is not totally in agreement with the study. This could be modified at “ *Samonella *diversity isolated from ... “ without the word “Prevalence” because the study is not a follow-up of previously reported cases in those same conditions

**Introduction**

 In the fourth line you would change the word “poisoning” by contamination

 At the end correct one “and” that is in italics

 When you say "among the layer hens, no information about prevalence or diversity of *Salmonella* has been reported", you could specify or support if that lack of information is only in Ecuador or at a South American or global level. In the same way, it could important to include some epidemiological data of salmonelosis in Ecuador.

**Methodology**

 I think that is not necessary to do DNA extraction to perform PCR, because *Salmonella* an is gram negative bacteria and during the start of the PCR where the temperature rises to 90 ° C it is possible to destroy cellular wall and release the DNA. However, it does not affect the results, taking into account that the procedure has been carried out correctly avoiding cross contamination.

 On the other hand, in order to reproduce the methodology of this study, the program used for PCR should be indicated. The concentration of primers (30 uM) is the final or initial concentration?

 It would be useful to indicate why those particular genes were used as molecular markers for the identification of the species and the serovars

**Results**

 You can ignore the phrase "amplification of the bcfC gene" and leave the table 1 referenced because it is a bit redundant

 Regarding statistical analysis and sample number in representation or not, I prefer not to give many opinions because I'm not qualified enough for that. However, it would be important that the methodology explain in detail how the statistical analysis was performed and the calculation of the incidence.

 In order of reference, table 1 of results should be table 2 and vice versa

 Regarding table 1 (positive S *almonella *samples ...), it could be a little more consistent with what they explain in the methodology because it took me a bit to understand it, taking into account the classification of columns in terms of location, samples, etc.

**Discussion**

 I believe that at some point it can be said that the regulations of Ecuador in relation to the health and control of layer farms and local markets and explain how the work is contributing to improve local control, taking into account that this layer farms produce around 60% of egg production in the country.

I have read this submission. I believe that I have an appropriate level of expertise to confirm that it is of an acceptable scientific standard, however I have significant reservations, as outlined above.
